# A Multicentric Analysis of a Pre-Ecographic Score in Pregnancy: Time for a Dedicated Classification System

**DOI:** 10.3390/epidemiologia6030039

**Published:** 2025-07-24

**Authors:** Gianluca Campobasso, Fabio Castellana, Annalisa Tempesta, Alice Bottai, Annachiara Scatigno, Elisa Rizzo, Francesca Petrillo, Grazia Cappello, Prisco Piscitelli, Roberta Zupo

**Affiliations:** 1 Division of Obstetrics and Gynecology, Vito Fazzi Hospital, 73100 Lecce, Italy; elisarizzo.lecci@gmail.com; 2 Department of Interdisciplinary Medicine, University of Bari Aldo Moro, 70100 Bari, Italy; castellanafabio@gmail.com (F.C.); zuporoberta@gmail.com (R.Z.); 3 Fetal Medicine Unit, Di Venere and Sarcone Hospitals, 70131 Bari, Italy; atempesta2000@yahoo.it; 4 Azienda USL Toscana Nord Ovest, 56121 Pisa, Italy; alibott92@alice.it; 5 Department of Obstetrics and Gynecology, IRCCS Foundation Policlinico San Matteo, 27100 Pavia, Italy; annachiara.scatigno@gmail.com; 6 Department of Clinical, Surgical, Diagnostic and Paediatric Sciences, University of Pavia, 27100 Pavia, Italy; 7 Policlinico Sant’Orsola Malpighi, Università Alma Mater Studiorum di Bologna, 40138 Bologna, Italy; francesca-pet@virgilio.it; 8 Ospedale Santa Maria Alle Scotte, Università Degli Studi di Siena, 53100 Siena, Italy; g.cappello@student.unisi.it; 9 Department of Psichology and Health Sciences, Pegaso University, 80143 Naples, Italy; prisco.piscitelli@unisalento.it

**Keywords:** ultrasonography, pregnancy, maternal risk factors, pre-echographic score, imaging quality, anomaly ultrasound scan

## Abstract

**Objectives**: The objectives are to evaluate the influence of different maternal characteristics on ultrasound image quality and operator satisfaction, and to assess, preliminarily, a rating scale to stratify the difficulty level of ultrasound examination in early gestation. **Methods**: A multicentric observational study of ultrasound scans was carried out on singleton pregnant women undergoing routine gestational ultrasound at 11–14 weeks and 19–21 weeks of gestation at two Prenatal Care Centers in the Apulia region (Southern Italy). Inclusion criteria included the presence of one or more limiting features, i.e., obesity, retroverted uterus, myomas, previous abdominal surgery, and limited echo-absorption. Each woman was given an overall pre-echographic limiting score from 0 to 9. The outcome measure was the operator’s satisfaction with the examination, scored on a Likert scale. Nested linear regression models (raw, semi- and fully adjusted) were built for each of the two trimesters on the pre-ecographic limiting score (0–9 points) as dependent variables, with the operator’s satisfaction as the regressor. **Results**: The whole sample included 445 pregnant women. The two-center samples did not show statistically different baseline features. The operator’s satisfaction with the sonographic examination was significantly (and inversely) related to the pre-echographic limiting score, regardless of the mother’s age, the operator performing the ultrasound, the Hospital Center where the ultrasound examination was performed, and the duration of the sonographic examination. **Conclusions**: A number of maternal conditions need to be monitored for good ultrasound performance; using a specific rating scale to stratify the level of difficulty of the ultrasound examination at early gestation could represent a potentially useful tool, although it requires further validation.

## 1. Introduction

Obstetric ultrasound is a noninvasive imaging tool that uses sound waves to scan the abdominal and pelvic cavity of the pregnant woman to provide timely and accurate information about fetal anatomy and skim structural abnormalities [1]. In 2016, the World Health Organization (WHO) first recommended ultrasound as a routine aspect of prenatal care [2]. Early in pregnancy, ultrasonography provides suitable tools to drive decisions to confirm viability, accurately establish gestational age, determine the number of fetuses, and, in the presence of multiple pregnancies, assess chorionicity and amnionicity [3]. Toward the end of the first trimester, scanning also provides an opportunity to detect major fetal abnormalities and, in health systems that offer first-trimester aneuploidy screening, to measure nuchal translucency (NT) thickness [4].

However, performing an obstetric ultrasound turns out to be a tricky practice because of the arduous fetal anatomy, the challenges posed by fetal movements, and the habitus of the maternal body. Moreover, several major malformations may develop later in pregnancy or may go undetected even with proper equipment and in the most experienced hands. The quality of ultrasound images may also be impacted by operator dependence in performing ultrasound examinations to some extent. These features add to the variability in the quality of the imaging procedure. Further, the objective imaging evaluation is weighted down by the variation in the locations where ultrasounds are performed and the substantial variability in the training of personnel performing and interpreting obstetric ultrasounds [5].

Contributing to the feasibility and quality of early pregnant routine ultrasonography lies a cluster of maternal conditions that are often poorly considered in clinical practice. Of these, greater research attention to date has been focused on the effect of maternal body mass index (BMI) on the image quality of routine fetal examinations [6,7]. A large British retrospective study [8] evaluated a total of 26,954 ultrasound images of 3251 women falling within different BMI ranges taken during routine mid-trimester ultrasound examinations. Using a large set of images acquired by many sonographers on multiple ultrasound machines, Yaqub and colleagues showed that the higher the maternal BMI category, the less likely the fetal images were to meet quality criteria. Such maternal features, along with any measurement biases related to the ability and experience of the operator, as well as factors such as amniotic fluid index (AFI), fetal weight, presentation, and sex, may add a major limitation to fetal imaging [9,10,11].

To date, further maternal features have been little studied from the perspective of their impact on image quality, and thus a large research window needs to be filled on this topic to allow key information to be disseminated among pregnant women in order to better address diagnostic trajectories led by routine scans of fetal anatomy. The present research sought to fill this gap by investigating the effect of a cluster of maternal limiting features on the feasibility and quality of ultrasound imaging using a multi-center convenience sample of first- and second-trimester pregnant women from Southern Italy.

The main goal of this research was to understand the effect of one or more limiting factors in influencing practitioner satisfaction and perceived quality during a prenatal ultrasound examination in order to infer its clinical and medico-legal relevance. Along these lines, we seek to prove the usefulness of having a rating scale available to stratify the level of difficulty of ultrasound examination at 11–14 and 19–21 weeks of gestation, since to date there are no studies that have used one or more limiting factors to create a difficulty scale for ultrasound examinations in pregnancy.

## 2. Material and Methods

### 2.1. Study Population and Design

This was a cross-sectional, multi-center observational study of ultrasound scans performed on singleton pregnant women who underwent ultrasound imaging at 11–14 weeks and 19–21 weeks’ gestation. From March 2023 to March 2024, a convenience cohort of pregnant women who presented for prenatal care at the Fetal Medicine and Prenatal Diagnosis Unit of “Di Venere” Hospital in Bari (Apulia, Southern Italy) and the Obstetrics and Gynecology Unit of Vito Fazzi Hospital in Lecce (Apulia, Southern Italy) to receive routine gestational ultrasound was recruited for the present study.

The study protocol (ClinicalTrials.gov Identifier: NCT06441929) met the principles of the Declaration of Helsinki [12] and was approved by the IRB of Vito Fazzi Hospital in Lecce (Apulia, Italy). All participants gave informed consent prior to enrollment following the Helsinki Declaration of 1964 and subsequent revisions.

The inclusion criteria were singleton pregnancy and the presence of one or more of the risk factors widely described in the literature as limiting features, i.e., excess weight (BMI > 24.9 Kg/m^2^) [13], retroverted uterus [14], the presence of myomas [15], a previous abdominal surgery [16], and a limited echo absorption [17]. No exclusion criteria related to women’s gestational age or ethnicity were applied.

### 2.2. Clinical and Instrumental Assessment

A team of experienced obstetricians and nurses collected baseline maternal information that included age at the time of gestation (years), anthropometric parameters (weight (Kg), height (cm), and body mass index (i.e, BMI, Kg/m^2^)), gestational age at the time of the ultrasound scan (weeks, days), time taken to obtain the abdominal ultrasound (minutes), the operator who performed the examination (gynecologist), the presence/absence of a retroverted uterus (expressed as a dichotomous variable), the presence/absence of myomas (expressed as a dichotomous variable), eco absorbency (expressed as good, poor, or very poor), and the presence/absence of previous abdominal surgery (expressed as a dichotomous variable). Tissue echosorbency was evaluated using a predefined three-level ordinal scale (good, poor, very poor), based on image-quality criteria and assessed by experienced operators. However, we acknowledge that this parameter retains an intrinsic degree of subjectivity, which represents a potential limitation of the study.

Furthermore, data were collected about the approach (abdominal or vaginal), the timing of the ultrasound examination (20 min, between 20 and 30 min, or >30 min), and whether the operator was required to perform 1, 2, or >2 attempts to complete the examination.

When data collection was completed, each pregnant woman was assigned a score based on the presence of one or more limiting factors. BMI was categorized using standard WHO thresholds: normal weight (18.5–24.9 kg/m^2^), overweight (25.0–29.9 kg/m^2^), obesity grade I (30.0–34.9 kg/m^2^), grade II (35.0–39.9 kg/m^2^), and grade III (≥40.0 kg/m^2^), corresponding to a score of 0 to 4 points in the pre-echographic scale. Echo absorption was rated using a three-level ordinal classification (good, poor, very poor) based on predefined image-quality criteria, and was assigned a score from 0 to 2. Each pregnant woman was given an overall pre-echographic limiting score from 0 to 9, indicating an increasing number of factors limiting the ultrasound examination. Further, to frame the level of the operator’s satisfaction with the examination, a rating of “low”, “good”, or “very good” was allocated to each subject based on a Likert scale.

As for the instrumental assessment, prenatal ultrasounds were performed by experienced physicians using the GE Voluson E10 Ultrasound System (General Electric Healthcare Ultrasound, Zipf, Austria) equipped with a 2–9 MHz wide-band convex probe (C2-9-D). Then, two qualified nurses, trained to conduct consistent clinical procedures, collected the anthropometric measurements with patients wearing light clothing and having bare feet. After overnight fasting, variables were collected simultaneously between 7 am and 10 am. Height was measured to the nearest 0.5 cm using a wall stadiometer (Seca 711; Seca, Hamburg, Germany). Body weight was determined to the nearest 0.1 kg using a calibrated scale (Seca 711; Seca, Hamburg, Germany). BMI was calculated by dividing body weight (Kg) by the square of height (m^2^) and was classified according to World Health Organization criteria as normal weight (18.5–24.9 Kg/m^2^), overweight (25.0–29.9 Kg/m^2^), grade I obesity (30.0–34.9 Kg/m^2^), grade II obesity (35.0–39.9 Kg/m^2^) and grade III obesity (≥40.0 Kg/m^2^) [18].

### 2.3. Statistical Analysis

The entire sample was divided according to the evaluating Hospital Center to obtain a descriptive analysis for the 1st semester (Table 1). Table 2 shows a description of the whole sample according to ecography satisfaction. The 2nd semester is presented in Table 3. Normal distributions of quantitative variables were tested using the Kolmogorov–Smirnov test. Therefore, data were reported as Mean ± Standard Deviations (M ± SD) and median (IQR) for continuous measures and frequency and percentages (%) for all categorical variables. Based on the distribution of quantitative data, a nonparametric approach was used to assess differences between groups, using the Wilcoxon Sum rank test for independent samples. A *p*-value less than or equal to 0.05 was considered significant. Two nested linear regression models were built for each of the two trimesters on the pre-ecographic limiting score (defined as described in the Methods section from 0 to 9 points) as dependent variables and with operator satisfaction as regressors.

Despite including reversed models where the pre-echographic score was treated as a predictor of operator satisfaction, we retained the original approach—modeling the score as the dependent variable—as our primary analytical strategy. This choice reflects the exploratory nature of the score construction: we aimed to understand whether operator satisfaction, a real-time and experience-based outcome, could reflect the presence and weight of known maternal limiting factors, and thus support the construct validity of the proposed scoring system. We acknowledge that reverse modeling may offer stronger predictive capabilities. Still, in this early stage of development, our primary goal was to test the score’s internal coherence and relation with clinically relevant outcomes.

Table 4 and Table 5 show the linear regression models for each assessment period, including a raw model, a semi-adjusted model (adjusted for mother’s age and Hospital Center), and a fully adjusted model (adjusted for mother’s age, Hospital Center, operator, and echographic assessment duration). In order to test homoscedasticity in models, the Breusch–Pagan test was adopted. For the purpose of assessing model performance, the fitting of the models was compared using the ANOVA test and evaluating the R2 and AIC parameters of the models (Supplementary Table S1). The VIF function of the cars Package was adopted to test multicollinearity for each linear regression model.

To further assess the relationship between pre-echographic score and operator satisfaction, we conducted ordinal logistic regression analyses, treating satisfaction as an ordinal dependent variable (low, good, very good). Three models were tested for both trimesters: a raw model, a partially adjusted model (adjusted for maternal age and Hospital Center), and a fully adjusted model (further including operator and scan duration). The results are reported in Supplementary Table S2.

Model performance was evaluated through Akaike Information Criterion (AIC), deviance, and pseudo-R^2^ indices (McFadden, Cox & Snell, and Nagelkerke) using IBM SPSS Statistics for Windows, version 29.0 (IBM Corp., Armonk, NY, USA). As shown in Supplementary Table S3, the fully adjusted models demonstrated the best fit across both trimesters, with lower AIC and deviance values, and higher explanatory power as indicated by all pseudo-R^2^ statistics. The statistical analysis was performed using RStudio 13.4 by a senior biostatistician (FC).

## 3. Results

The study population included a sample of 445 pregnant women (281 and 164 subjects from Bari and Lecce Hospital Centers, respectively) presenting at least one limiting factor at baseline (i.e., excess weight, myomas, previous abdominal surgery, reduced echo absorbency, and retroverted uterus) as inclusion criteria. Table 1 shows a description of the whole 1st trimester according to the Hospital Center (*n* = 298). According to the values of weight (Kg), BMI (Kg/m^2^), gestational age, presence of retroverted uterus (yes/no), presence of myomas (yes/no), operator’s ultrasound satisfaction grading (i.e., “low,” “good,” or “very good”), tissue echo absorption on ultrasound, echography duration, previous surgery (yes/no), mother’s age, and pre-echographic score, the samples from the two Hospital Centers showed no statistically significant differences. The only discrepancy showed the distribution of the type of ultrasound approach (transabdominal/transvaginal) between the two Centers (*p* < 0.03).

Table 2 presents the characteristics of the study population stratified by levels of operator satisfaction (low, good, very good) during first-trimester ultrasound examinations (*n* = 298). A significant association was observed between operator satisfaction and several maternal or procedural factors. Women in the “low satisfaction” group had significantly higher weight (mean 89.3 kg vs. 67.1 kg and 62.9 kg; *p* < 0.01) and BMI (mean 33.3 vs. 24.9 and 23.5; *p* < 0.01) compared to the “good” and “very good” groups. The pre-echographic score was also markedly higher in the low satisfaction group (mean 3.98 vs. 1.29 and 0.76; *p* < 0.01), confirming its correlation with perceived image quality. Significant differences were found for the presence of myomas (*p* < 0.01), retroverted uterus (*p* = 0.03), and previous abdominal surgery (*p* = 0.03), all more frequent in the low-satisfaction group. Echo absorption also varied significantly across satisfaction levels (*p* < 0.01): poor or very poor absorption (levels 1–2) was reported in 96% of low satisfaction cases, but only in 37% and 23% of the good and very good groups, respectively. Ultrasound duration was significantly longer in the low satisfaction group, with 25% of exams lasting > 30 min (*p* < 0.01), compared to just 1% in the “very good” group. No significant differences were observed in maternal age, gestational age, or Hospital Center distribution.

Table 3 shows a description of the entire second trimester according to Hospital Center (*n* = 147). Based on the values of weight (Kg), BMI (Kg/m^2^), presence of retroverted uterus (yes/no), presence of myomas (yes/no), operator’s ultrasound satisfaction rating (i.e., “low,” “good,” or “very good”), tissue resorption on ultrasound, the distribution of the type of ultrasound approach (transabdominal/transvaginal), previous surgery (yes/no), mother’s age, and pre-ecographic score, the samples from the two Hospital Centers showed no statistically significant differences. The only discrepancies were in gestational age (*p* < 0.01) and ultrasound duration (*p* < 0.01).

The linear regression models (Table 4 and Table 5) built on the pre-echographic score as dependent variable and with the operator’s satisfaction as regressor showed similar findings for the two trimesters. As for the first trimester (Table 4), in the raw model, a “good” operator satisfaction shows an average reduction in the pre-ecographic score with a β of −2.69 (−3.17 to −2.22, *p* < 0.01) compared to low; this value decreases further to −3.22 (−3.73 to −2.71, *p* < 0.01) for “very good” satisfaction. The association remains statistically significant in the semi- and fully corrected model with a β value of −2.53 (−3.01 to −2.05, *p* < 0.01) and −3.32 (from −3.92 to −2.72, *p* < 0.01) for “good” and “very good” satisfaction, respectively.

As for the second trimester (Table 5), in the raw model, a “good” operator satisfaction shows a mean reduction in the pre-ecographic score with a β of −2.89 (−3.59 to −2.19, *p* < 0.01) compared to low; this value decreases further to −3.15 (−4 to −2.3, *p* < 0.01) for “very good” satisfaction. The association remains statistically significant in the semi-corrected and fully corrected model with a β value of −3.14 (−3.86 to −2.43, *p* < 0.01) and −3.84 (−4.86 to −2.83, *p* < 0.01) for “good” and “very good” satisfaction, respectively.

Model performance comparison is presented in Supplementary Table S1 for both the first and second trimester analyses. In the first trimester, the fully adjusted model showed the best performance, with the lowest AIC (1114.64) and lowest residual sum of squares (RSS = 696.41), as well as the highest R^2^ (0.42) and adjusted R^2^ (0.41), indicating an improved model fit and explanatory power after including all covariates (mother’s age, Hospital Center, operator, and scan duration). In contrast, the raw model (unadjusted) had the highest AIC (1133.02) and lowest R^2^ (0.37), suggesting limited explanatory capacity when the covariates are not considered. In the second trimester, a similar trend was observed: the fully adjusted model again showed the best performance (AIC = 595.27; RSS = 430.47; R^2^ = 0.44; adjusted R^2^ = 0.40), followed by the partially adjusted and raw models. Notably, although the raw and partially adjusted models had comparable RSS values (509.87 and 510.3, respectively), the partially adjusted model slightly improved fit metrics. These results support the importance of including adjustment variables in the model, particularly when accounting for operator and center effects in ultrasound performance evaluation.

Supplementary Table S2 presents logit ordered regression models assessing predictors of operator satisfaction in both trimesters. In all models, a higher pre-echographic score was significantly associated with lower odds of operator satisfaction (OR range: 0.47–0.59, *p* < 0.001). This inverse association remained robust after adjusting for maternal age, Hospital Center, operator, and scan duration. In the **first trimester**, satisfaction was also significantly influenced by operator identity. In the fully adjusted model, Operator 2 showed a strong negative association (OR: 0.15, *p* < 0.001), while Operators 3 and 4 were positively associated with satisfaction (OR: 5.17 and 7.03, respectively). Long scan duration (>30 min) was linked to lower satisfaction (OR: 0.23, *p* < 0.001). Model fit improved from Model 1 to Model 3, as reflected in decreasing AIC values (531.80 to 416.92). In the **second trimester**, the association between a higher pre-echographic score and lower satisfaction remained significant (OR: 0.48, *p* < 0.001). Operator 2 again showed a strong positive effect (OR: 30.22, *p* < 0.001), and Operator 5 also emerged as significant (OR: 4.79, *p* = 0.04). The Lecce center was associated with lower satisfaction compared to Bari. Fully adjusted models demonstrated the best performance (AIC: 211.85). These results confirm the predictive validity of the pre-echographic score and highlight the importance of operator and center effects in influencing satisfaction levels.

Supplementary Table S3 reports model performance metrics for the logit ordered regressions on operator satisfaction across both trimesters. For both timepoints, the fully adjusted models showed the best performance, with the lowest AIC and deviance values, and the highest pseudo-R^2^ indicators. In the first trimester, the fully adjusted model had a McFadden R^2^ of 0.3771, Cox and Snell of 0.548, and Nagelkerke of 0.624—substantially higher than the raw (0.1621, 0.2893, 0.3294) and partially adjusted models. Similarly, in the second trimester, the fully adjusted model achieved the best fit, with a McFadden R^2^ of 0.3982, Cox and Snell of 0.5718, and Nagelkerke of 0.6489, clearly outperforming the raw and partially adjusted versions. These findings confirm that including covariates such as Hospital Center, operator, and scan duration improves the explanatory power and fit of the models.

## 4. Discussion

The present study aimed to provide evidence on the influence of a cluster of maternal limiting features on operator satisfaction and perceived quality of ultrasonography imaging in early pregnancy by using a multi-center convenience sample of 445 first- and second-trimester Apulian pregnant women. Further, we aimed at demonstrating the usefulness of having a grading scale available to stratify the level of difficulty of ultrasound examinations at 11–14 and 19–21 weeks of gestation. As main findings, the two samples from different Hospital Centers did not show statistically different baseline features, and we found that the operator’s satisfaction with the sonographic examination was significantly (and inversely) related to the pre-echographic limiting score (given by normal body weight and lower maternal BMI, the absence of a retroverted uterus, fewer myomas, good tissue echo-absorption on ultrasound, and fewer previous abdominal surgeries), regardless of the mother’s age, the operator performing the ultrasound, the Hospital Center where the ultrasound examination was performed, and the duration of the sonographic examination. The score retained significance in both cohorts in an overlapping way, proving to be an effective grading scale available to the operator to predict the degree of satisfaction with the ultrasound examination at 11–14 and 19–21 weeks of gestation. Further, a significant variation in operator satisfaction observed between Centers, especially in the second trimester, may reflect differences in operator experience, resource availability, or local organizational practices.

To date, there are no studies that have used one or more limiting factors to create a grading scale for ultrasound examinations in pregnancy. Some previous studies have focused on excess weight as the main maternal limiting factor on ultrasound quality in pregnancy. Dashe and colleagues [19] studied the effect of maternal obesity on ultrasound detection of abnormal fetuses, finding that a residual risk of abnormality after a normal ultrasound examination was increased with increasing BMI, from 0.4% among women with normal BMI to 1.0% among women with obesity. In addition, the detection of abnormalities was lower among women with pregestational diabetes than among those with other high-risk indications: 38% versus 88%, respectively. Uhden and colleagues [20] compared the prenatal detection of four congenital heart defects (CHDs) and the image quality of five corresponding ultrasound planes among women classified as obese, overweight, and normal weight. These authors showed that the rate of insufficient ultrasound images increased from 6.4% in normal-weight patients to 17.4% in obese women in the 108 CHD cases.

Gandhi and colleagues [21] estimated whether BMI affected the assessment of nuchal translucency or nasal bone during first-trimester ultrasound examination for aneuploidy risk assessment. The findings showed that increased BMI was significantly associated with inadequate assessment of nasal bone (3% vs. 12.7%), increased ultrasound examination time (15.23 ± 8.09 min vs. 17.01 ± 7.97 min), and increased need for transvaginal ultrasound examination (23% vs. 41.8%). In contrast, the presence of previous abdominal surgery was not significantly associated with the inadequacy of nasal-bone assessment (7.8% vs. 4.4%), the need for transvaginal ultrasound examination (33.6% vs. 28.6%), or a longer examination time (16.22 ± 8.6 min vs. 15.92 ± 7.8 min).

The areas of application of this pre-echographic grading scale may be different. In fact, the presence of one or more limiting factors, and thus the level of difficulty expressed according to a grading/numerical scale, could justify an incomplete or inadequate visualization of a certain anatomical structure, especially if it is small in size with regard to the thorny issue of medico-legal litigation for neglected fetal anomalies. In this context, regarding the risks associated with a maternal overweight status, a paper published by Paladini [22] suggests talking to patients and husbands, gently and sensitively pointing out the direct relationship between maternal BMI, an impaired acoustic window, and a consequent increased chance of missing important fetal abnormalities on ultrasound. Further, this report recommends including in the information sheet usually attached to the ultrasound report a detailed explanation that obesity, as well as scars from previous cesarean sections, twinning, and/or the presence of myomas, can cause a reduction in the detection rate of congenital anomalies due to acoustic impairment. In this scenario, pre-test counseling is of great importance. The addition of some limiting factors among those mentioned in the present article may further support the concept. These measures will not be able to prevent all lawsuits, but they may reduce their incidence, because parents’ awareness of the reduced resolution of the ultrasound examination is important in the mechanism of legal action.

As concluded in our findings, it may be necessary to make multiple attempts to adequately complete the examination, resulting in a greater loss of time. Furthermore, in the presence of one or more limiting factors, it may be necessary to use one or more TV approaches, thus justifying the discomfort caused to the patient. Therefore, appropriate pre-test counseling may be key.

## 5. Limitations

Some limitations of the study should be mentioned. The cross-sectional design, which does not allow causal inference to be drawn, leaves room for future prospective studies to corroborate our findings. Moreover, operator satisfaction was used as a proxy indicator of real-time sonographic image quality and examination feasibility. While this subjective measure reflects the clinician’s experience during the scan, we acknowledge that it may vary between operators and settings. In addition, the use of a convenience sample may have introduced selection bias, although standardized inclusion criteria and comparable populations across centers helped reduce this risk.

A further limitation lies in the arbitrary construction of the pre-echographic score, which—despite being based on well-recognized limiting factors for pregnant women—has not yet undergone external validation. We therefore propose future studies to refine and validate the score through larger, prospectively recruited cohorts and multivariate statistical approaches to improve its reliability and clinical applicability.

Also, data on amniotic fluid index (AFI) and fetal activity were not systematically recorded across centers and could not be included in the multivariable models. Both factors are known to influence acoustic-window quality and may act as potential confounders. Their absence may introduce residual bias and should be addressed in future studies through standardized recording and inclusion in analytical models.

No correction for clustering by center or operator was applied due to sample size constraints, although both variables were included in the adjusted models. This methodological limitation is acknowledged and suggests the need for more complex hierarchical models in future research.

Further, in this study, fibroids were recorded as a dichotomous variable (present/absent), without further stratification by location or size. This lack of granularity, due to inconsistent data collection across centers, prevented classification according to FIGO or dimensional criteria. Future studies should consider including standardized fibroid subtypes and echo-absorption metrics to better capture the individual contribution of each limiting factor to ultrasound image quality.

Finally, no a priori power calculation was conducted, since this was conceived as a pilot exploratory study. Nevertheless, to our knowledge, no previous research has investigated the combined effect of multiple maternal limiting factors on perceived ultrasound quality and operator satisfaction in early pregnancy. Existing studies have primarily focused on maternal BMI as a single predictor. We regard this study as a first step toward a broader understanding of how maternal characteristics may influence imaging feasibility, with the aim of enhancing prenatal screening and promoting informed counseling in women of childbearing age.

## 6. Conclusions

Enhancing ultrasound quality may enable timely and accurate information on fetal anatomy and structural abnormalities in early pregnancy. There is a need to better advise women before pregnancy about the outcomes associated with some limiting factors in order to achieve good ultrasound performance and optimal prenatal screening. In this context, our findings stressed a range of maternal pre-conditions (i.e., body weight, absence of retroverted uterus, fewer myomas, good ultrasound tissue uptake, and fewer previous abdominal surgeries) to be monitored for good ultrasound performance and we propose a rating scale to stratify the level of difficulty of ultrasound examination at 11–14 and 19–21 weeks’ gestation.

Given the exploratory nature of this study, our findings should be interpreted as preliminary. The proposed scoring system, although promising, has not yet undergone formal validation and should be tested in future prospective studies across broader and more diverse populations.

## Figures and Tables

**Table 1:** Description of the whole sample according to the Hospital Center (1st trimester). *n*: 298. All data are shown as Mean ± SD, median (iqr) for continuous variables and *n* (%) for categorical ones.

	Bari		Lecce		
	Mean ± SD	Median (iqr)	Mean ± SD	Median (iqr)	*p*-Value
Proportions (%)	181 (60.70)		117 (39.30)		
Height (cm)	163.972 ± 5.981	164 (8)	163.504 ± 6.633	164 (8)	0.59
Weight (Kg)	71.695 ± 19.948	66 (26.5)	68.687 ± 18.69	65 (21.3)	0.17
BMI (Kg/m^2^)	26.662 ± 7.24	23.916 (10.236)	25.591 ± 6.357	24.112 (6.732)	0.33
Week (n)	12.155 ± 0.432	12 (0)	12.154 ± 0.795	12 (0)	0.17
Days (n)	3.354 ± 1.734	4 (3)	3.179 ± 1.568	3 (2)	0.28
RV Uterus	7 (3.90)		2 (1.70)		0.49
Myomas	6 (3.30)		8 (6.80)		0.16
Echography Type					
TA	151 (85.80)		108 (92.30)		**0.03**
TA/TV	25 (14.20)		8 (6.80)		
TV	--		1 (0.90)		
Echography Satisfaction					
Low	36 (19.90)		28 (23.90)		0.70
Good	85 (47.00)		52 (44.40)		
Very Good	60 (33.10)		37 (31.60)		
Echo Absorption					
0	97 (53.60)		68 (58.10)		0.71
1	50 (27.60)		28 (23.90)		
2	34 (18.80)		21 (17.90)		
Echography Duration (min)					
≤20	107 (59.10)		70 (59.80)		0.98
≤30	56 (30.90)		36 (30.80)		
>30	18 (9.90)		11 (9.40)		
Previous Surgery	30 (16.70)		14 (12.10)		0.27
Pre-Echography Score	1.818 ± 2.08	1 (3)	1.513 ± 1.897	1 (3)	0.25
Mother’s Age (years)	33.649 ± 5.161	33.66 (6.24)	32.66 ± 5.897	33.85 (6.88)	0.25

Wilcoxon’s sum rank test for continuous variables and Chi squared/exact Fisher’s test for categorical ones. Bold: statistical significance.

**Table 2:** Description of the whole sample according to ecography satisfaction. *n*: 298. All data are shown as Mean ± SD, median (iqr) for continuous variables and *n* (%) for categorical ones.

	Low		Good		Very Good		
	Mean ± SD	Median (iqr)	Mean ± SD	Median (iqr)	Mean ± SD	Median (iqr)	*p*-Value
Proportions (%)	64 (21.50)		137 (46.00)		97 (32.60)		
Height (cm)	163.75 ± 5.88	164 (8)	164.04 ± 6.39	165 (8)	163.46 ± 6.30	164 (8)	0.78
Weight (Kg)	89.34 ± 18.94	87 (25.125)	67.11 ± 17.20	61 (20.1)	62.90 ± 14.45	61 (13)	**<0.01**
BMI (Kg/m^2^)	33.26 ± 6.48	32.169 (7.944)	24.91 ± 6.04	22.816 (7.327)	23.49 ± 5.07	22.59 (4.038)	**<0.01**
Week (n)	12.3 ± 1.05	12 (0)	12.1 ± 0.396	12 (0)	12.1 ± 0.378	12 (0)	0.24
Days (n)	3.44 ± 1.52	3 (3)		3 (3)	3.05 ± 1.60	3 (2)	0.23
RV Uterus	0 (0%)		8 (5.8%)		1 (1.0%)		**0.03**
Myomas	10 (16%)		2 (1.5%)		2 (2.1%)		**<0.01**
Echography Type							
TA	52 (81%)		121 (90%)		86 (91%)		0.16
TA/TV	12 (19%)		13 (9.6%)		8 (8.5%)		
TV	0 (0%)		1 (0.7%)		0 (0%)		
Echo Absorption							
0	3 (4.7%)		87 (64%)		75 (77%)		**<0.01**
1	21 (33%)		38 (28%)		19 (20%)		
2	40 (63%)		12 (8.8%)		3 (3.1%)		
Echography Duration (min)							
≤20	21 (33%)		80 (58%)		76 (78%)		**<0.01**
≤30	27 (42%)		45 (33%)		20 (21%)		
>30	16 (25%)		12 (8.8%)		1 (1.0%)		
Previous Surgery	16 (25%)		17 (12%)		11 (11%)		**0.03**
Pre-Echography Score	3.98 ± 1.77	4 (2.25)	1.29 ± 1.68	0 (2)	0.763 ± 1.38	0 (1)	**<0.01**
Mother’s Age (years)	34.70 ± 4.76	34.83 (6.615)	32.66 ± 6.03	33.62 (7.08)	33.16 ± 4.95	33.41 (6.29)	0.07
Hospital							
Bari	36 (56%)		85 (62%)		60 (62%)		0.70
Lecce	28 (44%)		52 (38%)		37 (38%)		

Wilcoxon’s sum rank test for continuous variables and Chi squared/exact Fisher’s test for categorical ones. Bold: statistical significance.

**Table 3:** Description of the whole sample according to the Hospital Center (2nd trimester). *n*: 147. All data are shown as Mean ± SD, median (iqr) for continuous variables and *n* (%) for categorical ones.

	Bari		Lecce		
	Mean ± SD	Median (iqr)	Mean ± SD	Median (iqr)	*p*-Value
Proportions (%)	100 (68.00)		47 (32.00)		
Height (cm)	162.86 ± 6.623	162.5 (9)	162.617 ± 5.944	162 (8)	0.83
Weight (Kg)	75.451 ± 25.918	67 (34.275)	75.474 ± 25.038	68.5 (30.65)	0.96
BMI (Kg/m^2^)	28.445 ± 9.832	25.45 (12.998)	28.378 ± 8.723	25.39 (10.565)	0.86
Week (n)	20.32 ± 0.51	20 (1)	17.149 ± 6.747	20 (0)	**<0.01**
Days (n)	3.08 ± 1.785	3 (2)	6.532 ± 6.639	5 (3)	**<0.01**
RV Uterus	1 (1.00)		3 (6.40)		0.06
Myomas	3 (3.00)		1 (2.10)		0.99
Echography Type					
TA	92 (92.00)		44 (93.60)		0.99
TA/TV	8 (8.00)		3 (6.40)		
Echography Satisfaction					
Low	24 (24.00)		22 (46.80)		<0.01
Good	46 (46.00)		23 (48.90)		
Very Good	30 (30.00)		2 (4.30)		
Echoabsorption					
0	46 (46.00)		29 (61.70)		0.10
1	36 (36.00)		9 (19.10)		
2	18 (18.00)		9 (19.10)		
Echography Duration (min)					
≤20	28 (28.60)		25 (53.20)		**<0.01**
≤30	43 (43.90)		9 (19.10)		
>30	27 (27.60)		13 (27.70)		
Previous Surgery	22 (22.00)		8 (17.00)		0.48
Pre-Echography Score	2.04 ± 2.361	1 (4)	1.851 ± 2.274	1 (4)	0.56
Mother’s Age (years)	34.48 ± 5.85	34.575 (8.077)	33.246 ± 5.486	32.3 (7.395)	0.19

Wilcoxon’s sum rank test for continuous variables and Chi squared/exact Fisher’s test for categorical ones. Bold: statistical significance.

**Table 4:** Linear regression model on pre-echography score as dependent variables and regressors (1st semester).

	Beta Coef.	Std. Err.	CI 95%	*p*-Value	Beta Coef.	Std. Err.	CI 95%	*p*-Value	Beta Coef.	Std. Err.	CI 95%	*p*-Value
(Intercept)	3.98	0.2	3.59 to 4.38	<0.01	3.99	0.64	2.73 to 5.25	<0.01	2.63	0.71	1.25 to 4.02	<0.01
Echography Satisfaction (Low)	−2.69	0.24	−3.17 to −2.22	<0.01	−2.71	0.24	−3.19 to −2.23	<0.01	−2.53	0.24	−3.01 to −2.05	<0.01
Echography Satisfaction (Good)	−3.22	0.26	−3.73 to −2.71	<0.01	−3.24	0.26	−3.74 to −2.73	<0.01	−3.32	0.31	−3.92 to −2.72	<0.01
Hospital Center (Lecce)					−0.42	0.19	−0.79 to −0.04	0.03	−0.32	0.18	−0.67 to 0.04	0.08
Mother’s Age (years)					0.01	0.02	−0.03 to 0.04	0.77	0.02	0.02	−0.01 to 0.05	0.25
Echography Operator (2)									0.19	0.32	−0.43 to 0.82	0.55
Echography Operator (3)									0.96	0.41	0.16 to 1.76	0.02
Echography Operator (4)									1.11	0.31	0.5 to 1.72	<0.01
Echography Operator (5)									−0.25	0.32	−0.87 to 0.37	0.44
Echography Operator (6)									−0.5	0.44	−1.37 to 0.37	0.26
Echography Operator (7)									0.62	0.34	−0.05 to 1.29	0.07
Echography Duration (≤30 min)									0.98	0.21	0.56 to 1.4	<0.01
Echography Duration (>30 min)									1.34	0.35	0.66 to 2.03	<0.01

**Table 5:** Linear regression model on pre-echography score as dependent variables and regressor (2nd semester).

	Beta Coef.	Std. Err.	CI 95%	*p*-Value	Beta Coef.	Std. Err.	CI 95%	*p*-Value	Beta Coef.	Std. Err.	CI 95%	*p*-Value
(Intercept)	4.02	0.28	3.48 to 4.57	<0.01	4.37	0.95	2.5 to 6.24	<0.01	4.36	0.97	2.46 to 6.26	<0.01
Echography Satisfaction (Low)	−2.89	0.36	−3.59 to −2.19	<0.01	−3.04	0.35	−3.74 to −2.35	<0.01	−3.14	0.36	−3.86 to −2.43	<0.01
Echography Satisfaction (Good)	−3.15	0.43	−4 to −2.3	<0.01	−3.58	0.45	−4.46 to −2.7	<0.01	−3.84	0.52	−4.86 to −2.83	<0.01
Hospital Center (Lecce)					−1.02	0.34	−1.69 to −0.34	<0.01	−0.82	0.35	−1.51 to −0.14	0.02
Mother’s Age (years)					0.01	0.03	−0.05 to 0.06	0.88	0.01	0.03	−0.05 to 0.05	0.95
Echography Operator (2)									0.82	0.54	−0.23 to 1.87	0.13
Echography Operator (3)									−0.41	0.46	−1.32 to 0.49	0.37
Echography Operator (4)									−0.43	0.66	−1.72 to 0.86	0.51
Echography Operator (5)									−0.47	0.61	−1.67 to 0.72	0.44
Echography Operator (6)									0.04	0.75	−1.44 to 1.51	0.96
Echography Operator (7)									−1.35	0.66	−2.64 to −0.06	0.04
Echography Duration (≤30 min)									0.18	0.43	−0.66 to 1.02	0.67
Echography Duration (>30 min)									0.66	0.5	−0.32 to 1.64	0.19

## Data Availability

All data supporting the findings of this study are available from the corresponding author (GC) upon reasonable request.

## Supplementary Materials

The following supporting information can be downloaded at: https://www.mdpi.com/article/10.3390/epidemiologia6030039/s1, Table S1: Linear regression model performance comparison according to adjustments. Table S2: Logit Ordered regression model on echography satisfaction as dependent variables and regressor (2nd Semester). Table S3. Logit ordered regression model performance comparison according to adjustments.
