# Supplementary material for: A Multicentric Analysis of a Pre-Ecographic Score in Pregnancy: Time for a Dedicated Classification System
Source: Epidemiologia (Basel). 2025 Jul 24;6(3):39. doi: 10.3390/epidemiologia6030039 (PMC12371941; doi:10.3390/epidemiologia6030039)
Supplement: Supplementary file 1 [file epidemiologia-06-00039-s001.zip › epidemiologia-3651665-supplementary.pdf]

Supplementary Table S1. Linear regression model performance comparison according to adjustments.

Model	AIC	RSS	Degrees of Freedom	R2	Adj R2
First Trimester					
Fully Adj.	1114.64	696.41	291	0.42	0.41
Partially Adj.	1131.93	747.97	293	0.38	0.37
Raw Model	1133.02	760.85	295	0.37	0.36
Second Trimester					
**Model**	**AIC**	**RSS**	**Degrees of Freedom**	**R2**	**Adj R2**
Fully Adj.	595.27	430.47	133	0.44	0.40
Partially Adj.	608.12	510.3	144	0.36	0.34
Raw Model	610	509.87	143	0.35	0.35

Supplementary Table S2. Logit Ordered regression model on Echography Satisfaction as dependent variables and regressor (2nd Semester).

	**Model 1 (AIC: 531.80)**				**Model 2 (AIC:532.72)**				**Model 3 (AIC: 416.92)**			
**A) I Trimester**	**OR**	**Stand.Err**	**CI.95.**	**p value**	**OR**	**Stand.Err**	**CI.95.**	**p value**	**OR**	**Stand.Err**	**CI.95.**	**p value**
Pre-Echography Score	0.53	0.07	0.46 – 0.61	**<0.001**	0.52	0.07	0.45 – 0.6	<0.001	0.47	0.09	0.39 – 0.56	**< 0.001**
Hospital Center (Lecce)					0.68	0.24	0.42 – 1.08	0.09	0.73	0.28	0.42 – 1.25	0.24
Mother's Age (years)					0.98	0.02	0.95 – 1.03	0.46	0.99	0.02	0.95 – 1.04	0.86
Echography Operator (2)					0.03	0.75	0.01 – 0.15	**<0.001**	0.15	0.47	0.06 – 0.38	**<0.001**
Echography Operator (3)					0.51	0.72	0.13 – 2.09	0.35	5.17	0.64	1.47 – 18.14	**0.02**
Echography Operator (4)									7.03	0.48	2.74 – 18.04	**<0.001**
Echography Operator (5)									0.35	0.45	0.15 – 0.85	**0.02**
Echography Operator (6)									0.08	0.73	0.02 – 0.36	**<0.001**
Echography Operator (7)									0.67	0.5	0.25 – 1.78	0.42
Echography Duration (<=30 min)									0.53	0.33	0.28 – 1.01	0.06
Echography Duration (>30 min)									0.23	0.56	0.08 – 0.7	**<0.001**

	**Model 1 (AIC: 266.57)**				**Model 2 (AIC:243.89)**				**Model 3 (AIC:211.85)**			
**B) II Trimester**	**OR**	**Stand.Err**	**CI.95.**	**p.value**	**OR**	**Stand.Err**	**CI.95.**	**p.value**	**OR**	**Stand.Err**	**CI.95.**	**p.value**
Pre-Echography Score	0.59	0.09	0.49 – 0.7	**< 0.001**	0.53	0.1	0.44 – 0.64	**<0.001**	0.48	0.12	0.38 – 0.6	**< 0.001**
Hospital Center (Lecce)					0.14	0.43	0.06 – 0.33	**<0.001**	0.2	0.49	0.08 – 0.52	**0.001**
Mother's Age (years)					1.03	0.03	0.97 – 1.09	0.35	1.02	0.03	0.95 – 1.09	0.56

Echography Operator (2)	30.22	0.71	7.49 – 121.99	**< 0.001**
Echography Operator (3)	1.31	0.61	0.39 – 4.32	0.66
Echography Operator (4)	1.86	0.84	0.36 – 9.75	0.46
Echography Operator (5)	4.79	0.78	1.04 – 22.03	**0.04**
Echography Operator (6)	0.88	0.92	0.15 – 5.34	0.89
Echography Operator (7)	1.08	0.85	0.2 – 5.74	0.92
Echography Duration (<=30 min)	2.07	0.57	0.68 – 6.33	0.20
Echography Duration (>30 min)	0.42	0.68	0.11 – 1.57	0.19

Supplementary Table S3. Logit ordered regression model performance comparison according to adjustments.

**I Trimester**	**AIC**	**Devianza**	**LogLik**	**McFadden**	**CoxSnell**	**Nagelkerke**
Raw model	531,8072	525,8072	-262,9036	0,1621	0,2893	0,3294
partially adj.	532,7223	522,7223	-261,3611	0,1671	0,2966	0,3377
Fully adj.	416,923	390,923	-195,4615	0,3771	0,548	0,624
**II Trimester**	**AIC**	**Devianza**	**LogLik**	**McFadden**	**CoxSnell**	**Nagelkerke**
Raw model	266,5764	260,5764	-130,28821	0,1563	0,2799	0,3189
partially adj.	243,8938	233,8938	-116,94688	0,2427	0,3994	0,4551
Fully adj.	211,8599	185,8599	-92,92995	0,3982	0,5718	0,6489
